# Virtual Reality–Augmented Physiotherapy for Chronic Pain in Youth: Protocol for a Randomized Controlled Trial Enhanced With a Single-Case Experimental Design

**DOI:** 10.2196/40705

**Published:** 2022-12-12

**Authors:** Laura E Simons, Courtney W Hess, Ellison S Choate, Amanda R Van Orden, Alexandra G Tremblay-McGaw, Maria Menendez, Derek B Boothroyd, Gomathy Parvathinathan, Anya Griffin, Thomas J Caruso, Jennifer Stinson, Amy Weisman, Timothy Liu, Kurt Koeppen

**Affiliations:** 1 Department of Anesthesiology, Perioperative, and Pain Medicine Stanford University School of Medicine Stanford, CA United States; 2 Quantitative Statistical Unit, Department of Medicine Stanford University School of Medicine Stanford, CA United States; 3 Department of Anesthesia and Pain Medicine The Hospital for Sick Children Toronto, ON Canada; 4 The Research Institute The Hospital for Sick Children Toronto, ON Canada; 5 Department of Rehabilitation Lucile Packard Children’s Hospital Stanford, CA United States; 6 California Rehabilitation & Sports Therapy Palo Alto, CA United States

**Keywords:** chronic pain, adolescents, physiotherapy, virtual reality, single-case experimental design, mobile phone

## Abstract

**Background:**

Chronic musculoskeletal (MSK) pain is a prominent health concern, resulting in pain-related disability, loss of functioning, and high health care costs. Physiotherapy rehabilitation is a gold-standard treatment for improving functioning in youth with chronic MSK pain. However, increasing physical activity can feel unattainable for many adolescents because of pain-related fear and movement avoidance. Virtual reality (VR) offers an immersive experience that can interrupt the fear-avoidance cycle and improve engagement in physiotherapy. Despite promising initial findings, data are limited and often lack the rigor required to establish VR as an evidence-based treatment for MSK pain.

**Objective:**

This trial evaluates physiorehabilitation with VR in adolescents with MSK pain. This protocol outlines the rationale, design, and implementation of a randomized controlled trial enhanced with a single-case experimental design.

**Methods:**

This study is a 2-group randomized controlled trial assessing the use of physiorehabilitation with VR in adolescents with MSK pain. The authors will collaborate with physical therapists to integrate VR into their standard clinical care. For participants enrolled in standard physiotherapy, there will be no VR integrated into their physical therapy program. Primary outcomes include physical function and engagement in VR. Secondary outcomes include pain-related fear and treatment adherence. Moreover, we will obtain clinician perspectives regarding the feasibility of integrating the intervention into the flow of clinical practice.

**Results:**

The pilot study implementing physiorehabilitation with VR demonstrated that high engagement and use of physiorehabilitation with VR were associated with improvements in pain, fear, avoidance, and function. Coupled with qualitative feedback from patients, families, and clinicians, the pilot study results provide support for this trial to evaluate physiorehabilitation with VR for youth with chronic MSK pain. Analysis of results from the main clinical trial will begin as recruitment progresses, and results are expected in early 2024.

**Conclusions:**

Significant breakthroughs for treating MSK pain require mechanistically informed innovative approaches. Physiorehabilitation with VR provides exposure to progressive challenges, real-time feedback, and reinforcement for movement and can include activities that are difficult to achieve in the real world. It has the added benefit of sustaining patient motivation and adherence while enabling clinicians to use objective benchmarks to influence progression. These findings will inform the decision of whether to proceed with a hybrid effectiveness-dissemination trial of physiorehabilitation with VR, serving as the basis for potential large-scale implementation of physiorehabilitation with VR.

**Trial Registration:**

ClinicalTrials.gov NCT04636177; https://clinicaltrials.gov/ct2/show/NCT04636177

**International Registered Report Identifier (IRRID):**

DERR1-10.2196/40705

## Introduction

### Background

Chronic musculoskeletal (MSK) pain in adolescence is a significant public health concern with median prevalence rates of 11% to 38% and 3% to 5% in adolescents experiencing significant pain-related disability [1,2], costing US $19.5 billion annually in the United States alone [3]. Notwithstanding the personal burden and persistent physical and economic consequences for families, chronic pain in adolescence can also predispose the development of adult chronic pain [2,4]. For adolescents and adults, functional restoration requires the progressive increase in physical activity [5-12] despite the presence of pain, with physiotherapy (PT) [13] being a critical element in guiding progression. Despite the well-documented importance of PT, daring to increase movement while in pain can be physically and emotionally unattainable. Fear of pain has been identified as a particularly salient influence on pain outcomes [14-17], at times hindering clinical improvement [18].

Virtual reality (VR) has the potential to facilitate breaking the cycle of fear of movement and avoidance during PT. VR enables the user to interact with a computer-generated environment that harnesses visual, audio, and tactile sensory inputs to provide an immersive experience to facilitate reaching therapeutic goals. Within the context of PT, access to a multisensory 3D experience can support patients in overcoming obstacles [19,20] that can seem insurmountable in the *natural* world, such as physical movement using the affected limb. Owing to increased market availability and declining costs, VR use in health care has rapidly increased in recent years.

Although most commonly applied in the context of acute pain relief [20,21], research conducted by our group has highlighted the potential for application in the realm of chronic pain rehabilitation [22-24]. We conducted a pilot feasibility study, which included the development and testing of several unique VR experiences for youth undergoing chronic pain treatment and rehabilitation [24]. Included in the pilot study were 17 youths with chronic pain who reported high levels of immersion, an important indicator of the level of engagement participants felt in their VR world. For adolescents with multi-session data (n=8), improvements in pain, fear, avoidance, and functional limitations were observed. These findings, coupled with qualitative feedback from patients, psychologists, physiotherapists, and occupational therapists, provide initial support for physiorehabilitation with VR as an acceptable, feasible, and potentially useful intervention for patients with chronic pain [24].

These findings are consistent with the extant literature, where VR has been suggested as an alternative to opioids with the therapeutic mechanisms centered on distraction [25,26]; neuromodulation of body perception [27]; and exposure to feared and, thus, avoided movements [22,23,28,29]. Moreover, VR can potentially enhance motivation and engagement during physical rehabilitation, facilitate repetitive motions, and incorporate real-time and longitudinal feedback for the patient and clinician [30-33]. Perhaps most exciting is the prospect of VR to engage several cortical and subcortical neuronal circuits that potentiate learning and recovery [34,35], with the potential for enhanced cortical reorganization [21,36]. Although most studies reflect proof-of-concept, feasibility, and pilot randomized controlled trial (RCT) studies, a recently published RCT of VR combined with exercise for adults with fibromyalgia demonstrated greater improvements in pain, fear of movement, fatigue, level of physical activity, and quality of life when compared with exercise alone [28].

Current VR studies lack the rigor and measurements over time that are critical for establishing VR as an evidence-based treatment for chronic pain rehabilitation. Moreover, the successful implementation of VR in practice requires further assessment. Initial findings suggest that clinicians find that VR supports individually tailored treatment, increases engagement in treatment, and improves the provider-client relationship, but clinicians also report persistent technology-related issues, adverse patient experiences of dizziness or headache with VR, and barriers associated with initial onboarding of the VR technology—namely, initial cost, lack of intuitive technology, need for training and technological support, and lack of staff to support implementation [37]. Altogether, the successful deployment of VR in chronic pain rehabilitation will require evidence coupled with a clear understanding of its feasibility and implementation challenges.

### Objectives

This study is a randomized controlled feasibility trial enhanced with a single-case experimental design (SCED) to compare physiorehabilitation with VR with standard PT implemented within routine clinical care. We will evaluate the functional outcomes of physiorehabilitation with VR and standard PT and characterize the feasibility of a future hybrid effectiveness-dissemination trial of PT rehabilitation treatment with VR in routine PT practice. The primary effectiveness outcome is physical function for the adolescent, and the secondary outcomes are pain-related fear and fear of movement. For feasibility, the primary outcomes are treatment acceptability, engagement, and implementation in routine clinical care, and secondary outcomes are adverse events with VR and treatment feedback from patients and clinicians.

## Methods

### Ethics Approval

This study was approved by Advarra, an external Institutional Review Board (IRB) for multisite studies, as well as by the IRB at Stanford University (eProtocol 63582). Procedures will follow the ethical standards of the IRB and the Helsinki Declaration of 1975 as revised in 2000. Informed consent and assent will be obtained from all participants. This study is registered at ClinicalTrials.gov (NCT04636177).

### Participants and Setting

Adolescent participants will be recruited from one of the collaborating outpatient rehabilitation sites: an outpatient PT center within an academic medical center (eg, Stanford Children’s Health) or a private outpatient PT center (eg, California Rehabilitation and Sports Therapy and Agile Physical Therapy). Adolescents are eligible to participate if they (1) have localized or diffuse MSK pain [38,39], (2) are aged between 10 and 17 years, and (3) are proficient in the English language. Adolescents are ineligible to participate if they have (1) pain because of acute trauma (eg, active sprain, fracture, or surgery); (2) significant cognitive impairment; (3) significant psychiatric diagnoses that would interfere with treatment or VR use, such as active psychosis or suicidality; or (4) a condition that interferes with VR use, including history of seizure, facial injury precluding safe placement of headset, visual impairment, and significant hearing impairment affecting the ability to follow audio instructions, as extracted from the medical record and confirmed by the referring clinician.

### Recruitment

Adolescents who meet the eligibility criteria and their caregivers are informed of the study by their clinicians when they present to one of the clinical recruitment sites for their initial PT evaluation. In addition, a study flyer is posted on a bulletin board of all active clinical studies in the patient waiting room at each recruitment site. Clinicians and the flyer direct interested adolescents and caregivers to fill out an eligibility web-based screening form, which allows the research team to contact the family directly. If the eligibility criteria are met, a research coordinator contacts the family for additional screening and to complete the assent and consent process.

### Study Design

**Figure 1:**
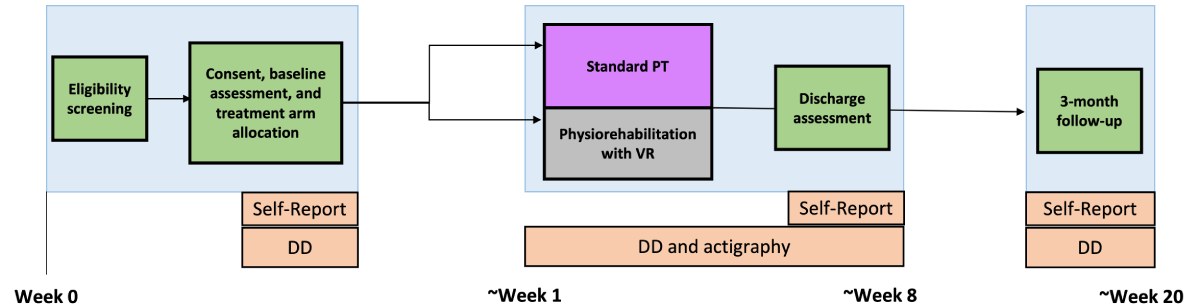
Study flow depicting eligibility, consent, baseline, discharge, and 3-month follow time points. DD: daily diary; PT: physiotherapy; VR: virtual reality.

This is a 2-group RCT enhanced with SCED using multiple measures. In single-case experiments, a participant is observed repeatedly at different levels of at least one independent variable, for example, assessing engagement with PT exercises throughout pretreatment baseline and across treatment. To accomplish this, adolescents will complete daily diaries during a pretreatment phase (estimated to range from 7 to 14 days in duration) and daily diaries from day 0, when VR headsets are distributed, to end of treatment (day 0+N) and for 7 days at the 3-month follow-up. Upon arrival at the first treatment session after the baseline assessment, adolescents are informed if they are assigned to physiorehabilitation with VR or standard PT. The treatment phase consists of an estimated 6 to 8 PT sessions of 1 hour, with the number and frequency of treatment sessions determined at the discretion of the clinician. In total, the study is expected to be completed over the course of 32 months. A total of 24 months are dedicated to study enrollment, randomization, and completion of the intervention. An additional 8 months are dedicated to completing 3-month follow-up assessments and data analysis (Figure 1).

### Rationale for Study Design

RCTs provide robust estimates of the between-subject treatment response (the average difference between the 2 groups) but do not provide sufficient data on how a specific individual responds to a given treatment because of heterogeneity in treatment effects. That is, an individual patient in an RCT could show no improvement, have an adverse reaction to treatment, or benefit from the active comparator even if the active comparator is shown to be statistically inferior. Although subgroup analyses are now encouraged to better elucidate differences in treatment responses between individuals, they require large cohorts of patients for sufficiently powered analyses. For specialized patient groups such as youth experiencing chronic pain, obtaining sufficiently large cohorts for mediation and moderation analyses within the confines of RCTs is often not feasible. SCED allows for the collection of statistically rigorous data at the level of the individual patient. Moreover, such data can be used in meta-analyses of individual effect sizes and multilevel modeling to provide group-level results from small and distinctive cohorts.

### Randomization

Randomization schemes are developed and maintained by the study statistician, DB. Once enrolled, participants are randomized to either physiorehabilitation with VR or standard PT and 2×3 stratified on fear or disability—high fear or high disability (empirically validated clinical cutoff scores of 26-40 on the Fear of Pain Questionnaire-Short Form [FOPQ-SF] [40] *or* 30-60 on the Functional Disability Inventory [FDI] [41], respectively) *or* low or moderate fear *and* low or moderate disability (FOPQ-SF ≤25 *and* FDI ≤29)—and pain site (upper and trunk *or* lower *or* diffuse). A block randomization strategy is used with randomly generated blocks of 2 and 4 to ensure near-equal distributions across arms and minimize the probability of predicting the next assignment. The study biostatistician creates separate randomization lists for each of the 6 strata before the start of patient recruitment, with each list long enough to include the total planned study size. A series of block sizes (either 2 or 4, with probability weights of two-thirds and one-third, respectively) is randomly created and, within each block, half is randomly assigned to physiorehabilitation with VR and the other half to standard PT. Copies of the randomization lists are kept by the biostatistician and research coordinator and not shared with other members of the team.

### Intervention Procedures

#### PT Procedure

All participants engage in a full course of PT for MSK pain that is delivered by a trained and licensed physiotherapist. PT sessions are individually tailored based on the Guide to Physical Therapist Practice 3.0, consisting of (1) therapeutic exercise (strengthening and endurance exercises), (2) neuromuscular re-education (balance and proprioception exercises), (3) therapeutic activities (functional performance and activities of daily living), and (4) use of modalities (heat or cold packs). All adolescents receive a home exercise program (HEP) as part of the standard PT treatment. The full course of PT includes approximately 6 to 8 PT sessions delivered over 6 to 12 weeks. An adequate dose of treatment is considered to be 75% completion of the prescribed sessions. The number of treatment sessions varies based on patient presentation and individualized treatment goals as determined by the clinician and research team. Patients can continue any treatments they are currently involved in. If they choose to initiate a new treatment after PT treatment begins, they are asked to notify the research team immediately as it may affect their involvement in the study.

#### VR Procedure

All participants receive a VR headset to use for the duration of treatment. The research team provides participants with an Oculus Quest 2 (Oculus) VR headset and orients the adolescents to its functionality.

#### Standard PT

For standard PT participants, the VR headset is preloaded with distraction-based games for recreational use at home until treatment is completed (Table 1). Clinicians do not discuss VR headset use with the standard PT participants. The research team provides an orientation session regarding the VR headset functions and preloaded games.

**Table 1:** Standard physiotherapy rehabilitation virtual reality (VR) game content.

Game	Description	Distraction type
Color Space^a^	Color in pieces of beautiful art and environments in virtual reality.	Creative design
Cubism^a^	Challenge the mind by solving deceptively simple puzzles and assembling increasingly complex shapes out of colorful blocks.	Puzzle game
Vacation Simulator^a^	Find optimal relaxation and efficient memory making while being at home.	Simulation
NatureTrek VR^a^	Explore tropical beaches, underwater oceans, and >20 different animals. Command the weather, take control of the night, or shape your own world.	Exploration
Star Chart^a^	Explore the Solar System, view constellations, and watch meteor showers. Stand on the moon; explore Mars with the Curiosity rover; and hold planets, moons, and stars.	Exploration
Wander^a^	Teleport to almost anywhere in the world, from the London Bridge to the Great Pyramids of Egypt.	Exploration

^a^Denotes game accessible through a public app store.

#### Physiorehabilitation With VR

Physiorehabilitation with VR participants bring their VR headset to each PT appointment, and a portion of the session can be delivered in VR. Participating clinicians are told that the goal for VR use in a session is at least 8 minutes, but they are also given clinical decision-making power to use VR more or less as they determine clinically feasible. Physiorehabilitation with VR engages participants in a series of immersive games customized and chosen to allow for a progressive increase in standing endurance and support individual PT goals (Table 2). Importantly, this trial does not test a specific VR game or program but the broad implementation of VR in clinical practice. Games implemented for this purpose may include Fruity Feet (Stanford Chariot Program), Alien Defense (Stanford Chariot Program), Beat Saber (Beat Games), and Tilt Brush (Google), among others. Physiotherapists discuss VR headset use with physiorehabilitation with VR participants and actively incorporate VR activities into the HEP. The pediatric pain rehabilitation team at Stanford Children’s Health worked collaboratively with the Stanford Chariot Program to develop physiorehabilitation with VR content [24]. Fruity Feet was developed using a user-centered approach with patient and clinician end-user feedback across four phases: (1) needs assessment, (2) prototyping, (3) iteration and refinement, and (4) feasibility and acceptability. It was designed to be developmentally appropriate for youth by focusing on fun while leaning on stylized graphics and encouraging in-game feedback. Gameplay mechanics were built around PT movement goals, for example, multiplanar stepping (ie, forward, side, and back), stomping, marching, kicking, raising legs to different heights, and active ankle range-of-motion tasks for lower extremities. Importantly, it was also built to scale to a patient’s mobility, ensuring that patients of all abilities could play the game and benefit from the VR intervention. Consistent with the recommendations for VR clinical trial methodology, this user-centered iterative design process yielded a program that, in addition to gamification, provides back-end mechanics, giving PT clinicians the capability to control intensity, affected side or extremity emphasis, mirroring, and movement exaggeration to leverage the potential neuromodulatory effects of VR coupled with targeted pain PT.

**Table 2:** Physiorehabilitation with virtual reality (VR) game content.

Game	Description	Rehabilitation engagement
Alien Defense Foot Cannon^a^	Squashing descending aliens before time runs out	Leg and hip strengthening with light cardiovascular exercise
Fruity Feet^a^	Stomping and kicking falling fruits and vegetables before time runs out	Leg and hip strengthening with light cardiovascular exercise
Alien Defense Slingshot^a^	Destroying aliens using the arms as a slingshot	Arm and shoulder strengthening
Bait!^b^	Fishing and passing time in front of water	Arm and shoulder strengthening
Fruit Ninja^b^	Slicing fruit that is thrown the player’s way	Arm and shoulder strengthening
Beat Saber^b^	Slicing blocks and dodging obstacles to the beat of the music	Arm, shoulder, leg, glute, and hip strengthening with cardiovascular exercise
BOX VR^b^	Punching and dodging obstacles to the beat of the music	Arm, shoulder, leg, glute, and hip strengthening with cardiovascular exercise
Dance Central^b^	Dance battling computerized opponents in various locations to popular songs	Cardiovascular exercise and full-body strengthening through dancing
OhShape^b^	Holding fun poses and dodging obstacles to the beat of the music	Arm, shoulder, leg, glute, and hip strengthening with light cardiovascular exercise and static holds
Pro Putt^b^	Playing VR golf	Arm swings and hip rotations
Racket Fury: Table Tennis^b^	Playing VR table tennis	Arm, shoulder, leg, glute, and hip strengthening with light cardiovascular exercise
Racket NX^b^	Hitting a ball against a 360 dome with a racket	Arm, shoulder, leg, glute, and hip strengthening with cardiovascular exercise
Space Burgers 2^a^	Racing through exciting, food-filled galaxies with optional stationary bicycle	Cardiovascular exercise through stationary biking or running arm motion
Synth Riders^b^	Following the targets as they lead the player through dance moves to popular songs	Cardiovascular exercise and full-body strengthening through dancing
Tilt Brush^b^	Drawing and painting in a 3D space	Arm and shoulder strengthening

^a^Denotes games accessible through Invincikids.

^b^Denotes games accessible through a public app store.

### Assessment of Outcomes

#### Overview

Adolescents and caregivers complete baseline, discharge, and 3-month follow-up assessments. The adolescent completes daily diaries, and the caregiver completes weekly health cost diaries from the date of consent until the end of treatment at discharge. An additional 7 daily diaries by the adolescent and 1 additional health cost diary by the caregiver are completed at the start of the 3-month follow-up. All adolescent and caregiver surveys are completed on the web through the secure web-based app REDCap (Research Electronic Data Capture; Vanderbilt University), and the diaries are completed through the mobile phone–based app LifeData (LifeData, LLC) [42]. Baseline and discharge assessments are completed in person or on the internet, with the 3-month follow-up completed on the internet. Table 3 details the outcomes, measure names, respondents, and time of assessment.

**Table 3:** Outcomes (primary, secondary, additional, single-case experimental design, exploratory, and implementation) and covariates.

Category, subcategory, and measure			Respondent	Time point administered^a^			
				0	1	2	3
**Effectiveness**							
	**Primary outcomes: physical function**						
		Lower Extremity Functional Scale	Adolescent	✓		✓	✓
		Upper Extremity Functional Index	Adolescent	✓		✓	✓
	**Secondary outcomes: pain-related fear and avoidance**						
		Fear of Pain Questionnaire-Short Form	Adolescent	✓		✓	✓
		Photographs of Daily Activities for Youth	Adolescent	✓		✓	✓
		Tampa Scale for Kinesiophobia-17	Adolescent	✓		✓	✓
**Feasibility**							
	**Primary outcomes: treatment acceptability and engagement**						
		Treatment satisfaction questionnaire	Adolescent			✓	✓
		Virtual Reality acceptability questionnaire	Adolescent^b^			✓	
		Virtual Reality acceptability questionnaire	Clinician^b^			✓	
		Pittsburgh Rehabilitation Participation Scale	Clinician		✓		
		ManageXR Usage data	Researcher		✓		
	**Secondary outcomes: treatment expectations, feedback, and adherence**						
		Treatment Expectancy and Credibility: Youth	Adolescent	✓			
		Semistructured interview	All			✓	
		Home exercise program	Clinician		✓		
		VR^c^ adverse events survey	Clinician		✓		
		ManageXR usage data	Researcher		✓		
		Percentage of dropouts	Researcher		✓		
		Tracked adherence to treatment and surveys	Researcher		✓		
**Additional **							
	**Outcomes: multiple**						
		Functional Disability Inventory	Adolescent	✓		✓	✓
		PROMIS^d^ Pediatric Mobility scale	Adolescent	✓		✓	✓
		PROMIS Pain Interference	Adolescent	✓		✓	✓
		Pain Catastrophizing Scale: Youth	Adolescent	✓		✓	✓
		Pain Intensity Numeric Rating Scale	Adolescent	✓		✓	✓
		Pain Self-Efficacy Scale: Youth	Adolescent	✓		✓	✓
		Patient Global Impression of Change	Adolescent			✓	✓
		Presence Measure: Youth	Adolescent			✓	
**Single case experimental data**							
	**Outcomes: engagement self-efficacy, distraction, function, and pain**						
		Daily diary	Adolescent	✓	✓		✓
**Exploratory**							
	**Outcomes: physical activity and health-related costs**						
		Modified Borg Dyspnea Scale	Clinician		✓		
		Physical assessment	Adolescent	✓		✓	
		ActiGraph tracked physical activity levels	Researcher	✓		✓	
		Health cost diary	Caregiver	✓	✓	✓	✓
**Covariates**							
	**Outcomes: demographics and pain history**						
		Demographic survey	Caregiver	✓			
		Medical history survey	Caregiver	✓			

^a^0=baseline, 1=discharge, 2=assessed throughout treatment, either daily (adolescents), weekly (caregivers) or after each physiotherapy session (clinicians), and 3=3-month follow-up.

^b^Only completed by those in physiorehabilitation with virtual reality (VR) arm.

^c^VR: virtual reality.

^d^Measures that are assessed throughout treatment, either daily or after each physiotherapy session.

^e^HEP: home exercise program.

^d^PROMIS: Patient-Reported Outcomes Measurement Information System.

#### Baseline Assessment

Once eligibility is confirmed, the baseline assessment is completed in 2 parts. First, adolescents and caregivers complete consent and baseline self-report questionnaires. Adolescents and caregivers are oriented to the mobile diaries, which adolescents complete daily and caregivers complete weekly while enrolled in treatment. Following the baseline study visit, adolescents and caregivers undergo a pretreatment data collection period for the number of days between consent and their subsequent PT appointment. During this time, adolescents complete daily diaries, and caregivers complete weekly health cost diaries. The second part of the baseline assessment takes place at the adolescent’s next PT session, during which a research coordinator completes a baseline physical assessment via ViFive (ViFive, Inc) [43], a motion capture app, consisting of the 6-minute Walk Test, Single Leg Balance Test, and Closed Kinetic Chain Upper Extremity test, and the adolescent receives an ActiGraph watch (ActiGraph, LLC) that monitors sleep and activity level throughout the trial.

#### Discharge Assessment

The discharge assessment occurs after all PT treatment sessions are completed. The discharge assessment consists of a physical assessment at the final session as well as self-report questionnaires. The diaries conclude, and the adolescents return the ActiGraph watch as well as the VR headset. Following completion of the study, adolescents and caregivers in the physiorehabilitation with VR group complete Zoom (Zoom Video Communications)-based or in-person semistructured interviews to provide feedback regarding their treatment experience. Finally, clinicians complete an acceptability measure assessing their experiences integrating VR into patient sessions for each participant in the physiorehabilitation with VR arm and, subsequent to discharge of their final trial patient, will complete Zoom-based semistructured interviews to further assess PT perceptions of feasibility in integrating VR.

#### Follow-up Assessment

The follow-up assessment occurs at 3 months after discharge, at which time adolescents complete a set of self-reported questionnaires as well as 7 additional daily diaries. Caregivers complete 1 additional health cost diary. Adolescents receive the battery of self-report questionnaires via REDCap and respond to the daily diaries via LifeData. Caregivers receive their single health cost diary via REDCap.

#### Process Assessment

Following each PT session, the adolescent’s clinician completes a brief series of questions regarding the adolescent’s engagement in PT and exercise exertion as well as any adverse events that occurred during the session. Clinicians document and describe the HEP they prescribed to the adolescent to be completed between sessions.

### Effectiveness Outcomes

The primary effectiveness outcome is physical function (adolescent), measured using the Lower Extremity Functional Scale (LEFS) and Upper Extremity Functional Index (UEFI). The secondary effectiveness outcome is pain-related fear and avoidance, measured using the FOPQ-SF, the Photographs of Daily Activities for Youth (PHODA-Youth), and the Tampa Scale for Kinesiophobia-17 (TSK-17).

#### LEFS Measure

The LEFS is a 20-item self-report survey that asks participants to report on their ability to perform everyday tasks using their lower extremities [44]. Participants rate the level of difficulty associated with a range of everyday activities on a 5-point Likert scale (0=“extreme difficulty/unable to perform activity” to 4=“no difficulty”). Summed scores indicate the level of function, with higher scores indicating more functionality.

#### UEFI Measure

The UEFI is a 20-item self-report survey that asks participants to report on their ability to perform everyday tasks using their upper extremities [45]. Participants rate the level of difficulty associated with a range of everyday activities on a 5-point Likert scale (0=“extreme difficulty/unable to perform activity” to 4=“no difficulty”). For both the LEFS and UEFI, scores are summed, with a maximum score of 80 and where higher scores indicate better functioning. A minimum level of detectable change (confidence level=90%) is defined as a ≥9-point change based on the existing literature on patients with pain [46-48].

#### FOPQ-SF Measure

The FOPQ-SF consists of 10 items, with each item rated on a 5-point Likert scale (0=“strongly disagree” to 4=“strongly agree”) [49]. The FOPQ-SF contains questions assessing both fear of pain and avoidance of activities in the context of pain. The total score is derived by summing the items, with higher scores indicating greater pain-related fear and avoidance of activities.

#### PHODA-Youth Measure

The PHODA-Youth is a 50-item measure assessing worry associated with activities of daily living (13 items), sports or exercise activities (15 items), school or social activities (13 items), and upper extremity activities (9 items) [50]. To complete each item*,* the patient is exposed to a photograph and label of the activity and asked to rate their worry “that this activity would be harmful to your pain” by dragging each photograph along a “worry thermometer” ranging from 0 to 10. Each photograph is given a rating according to its position on the thermometer. Patients then rate their anticipated pain if they engaged in the activity. The mean perceived harm and anticipated pain scores (ranging from 0 to 10) are calculated as the sum of each rating divided by the total number of pictures.

#### TSK-17 Measure

The TSK-17 is a 17-item self-report measure assessing fear of movement that has been implemented in a variety of pain conditions [51], including youth with chronic pain [52,53]. The 2 subscales assess activity avoidance and somatic focus, with higher total and subscale scores indicating a greater fear of movement, activity avoidance, and somatic focus.

### Feasibility Outcomes

The primary feasibility outcomes are treatment satisfaction, acceptability, and engagement. The secondary feasibility outcomes are treatment expectations, treatment feedback, treatment fidelity, treatment adherence and retention, and adverse events.

#### Treatment Satisfaction

Treatment satisfaction is assessed using an adapted version of the Pain Service Satisfaction Test. The Pain Service Satisfaction Test is a 22-item measure that asks patients about their experiences in pain treatment, for instance, perceptions of the effectiveness of the intervention, the treatment team, and the impact on their own outcomes [54]. Scores are summed based on responses, with higher scores indicating greater satisfaction with treatment.

#### Acceptability

To evaluate the acceptability of the treatment intervention, the VR acceptability questionnaire is an 11-item measure completed by patients and clinicians at discharge. For patients, it asks about their enjoyment of VR, satisfaction with the VR treatment, perceived reduction of pain, and barriers experienced during the physiorehabilitation with VR intervention. For clinicians, it assesses their perceived difficulty associated with VR use, assessment of the games, ability of VR to facilitate engagement, overall satisfaction levels, and willingness to implement VR in the future for those in the physiorehabilitation with VR arm.

#### Treatment Engagement

**Figure 2:**
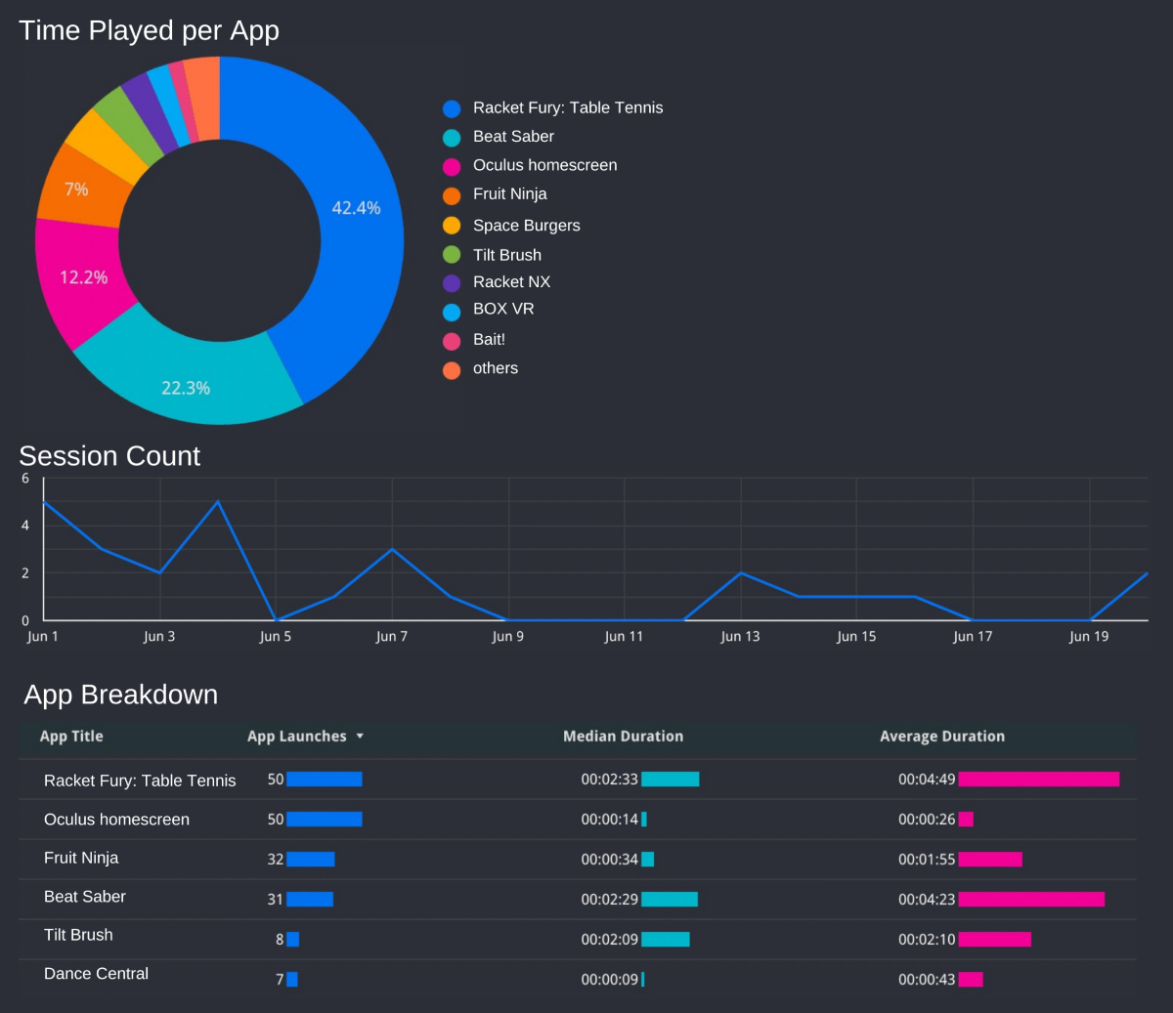
Oculus analytics dashboard via ManageXR software (Mighty Immersion, Inc) capturing virtual reality engagement.

To assess patient engagement in the physiorehabilitation with VR intervention, clinicians complete a postsession survey following every session, which includes the Pittsburgh Rehabilitation Participation Scale [55]. Clinicians rate the perceived patient’s motivation. Engagement is rated on a 6-point Likert scale (1=“none” to 6=“excellent”). Clinicians do not fill out a survey if the patient did not attend their session. Clinicians are instructed to select a lower rating when in doubt, for instance, “good” rather than “very good.” In addition, VR use is tracked via the ManageXR software (Mighty Immersion, Inc) preloaded onto each VR headset. Analytics regarding which VR games are played, duration of play per game, total play duration, and the number of application launches are displayed in the Oculus dashboard (Figure 2). The Oculus dashboard will be used to assess engagement in VR and fidelity to VR use for physiotherapy and HEPs.

#### Treatment Expectancy

Treatment expectations are measured using the child Treatment Expectancy and Credibility measure (TEC-C) [56]. The TEC-C comprises 6 items assessing expectations related to the effectiveness of the current treatment. The TEC-C is completed by the patient after the first treatment session.

#### Treatment Feedback

Patient and clinician feedback regarding the VR intervention is collected via semistructured interviews. The goal of the interviews is to better understand the experience of using VR, strengths of the VR intervention, and barriers or considerations for future use.

#### Treatment Fidelity

Treatment fidelity is assessed by examining VR use analytics displayed on the ManageXR dashboard (Figure 2). For the physiorehabilitation with VR arm, clinicians aim for at least 8 minutes of the session to be dedicated to VR exercises and, therefore, the number of sessions reaching this benchmark can be tracked. In addition, at least 15 minutes of the HEP include VR in the physiorehabilitation with VR arm. HEPs across both treatment arms are requested in the clinician postsession survey and, for the physiorehabilitation with VR arm, we can compare with VR use tracked in the ManageXR software.

#### Treatment Adherence and Retention

Adherence and retention are assessed by examining patient adherence to daily diaries, the percentage of patients who drop out before treatment completion, and the percentage of sessions completed on schedule.

#### Adverse Events

The clinician tracks adverse events related to VR use. Specifically, in the postsession survey, clinicians can indicate any adverse events of dizziness, nausea, or disorientation that occurred. As part of clinician orientation to the VR, important safety precautions are discussed to reduce the potential for accidents to occur.

### Additional Outcomes

Additional outcomes of interest include changes in functional disability, mobility, pain interference in life, pain catastrophizing, pain intensity, self-efficacy while in pain, global impression of change, and perceived immersion in the VR technology. Table 3 details the outcomes, measure names, respondents, and time of assessment.

#### Functional Disability

Functional disability is assessed using the FDI, a 15-item self-report measure of perceived difficulty in performing activities in the school, home, physical, and social contexts*.* Items are rated on a 5-point Likert scale (0=“no trouble” to 4=“impossible”) [57]. Items are summed to obtain a total score, with higher scores indicating greater disability. The FDI is widely used in pediatric pain research and is recommended as the gold-standard measure of physical functioning in school-age children and adolescents for clinical trials in pediatric chronic pain.

#### Mobility

Mobility is assessed using the Patient-Reported Outcomes Measurement Information System (PROMIS) Mobility measure, a subset of the PROMIS physical health function self-report outcomes [58]. This test is typically used in adult and pediatric populations with chronic conditions. The Mobility subscale measures perceived capabilities related to mobility tasks such as getting up from a chair or running. The PROMIS Mobility items are written in the past tense (eg, “I could...”), all use a standard recall of “in the past 7 days,” and have a 5-point Likert scale (0=“not able to do” to 4=“with little to no trouble”). Scores are summed, with higher scores indicating greater mobility.

#### Pain Interference

The PROMIS Pain Interference instrument assesses patient perception of the impact of pain on their ability to engage across several life domains—namely, social, emotional, and recreational activities [58]. The PROMIS Pain Interference items use a standard recall of “in the past 7 days” with items such as “it was hard to have fun when I had pain.” Responses are provided on a 5-point Likert scale (0=“almost never” to 4=“almost always”). PROMIS Pain Interference items are summed, with higher scores indicating more pain-related interference in a patient’s life.

#### Pain Catastrophizing

The Pain Catastrophizing Scale-Children assesses negative cognitions associated with pain [59]. The Pain Catastrophizing Scale-Children comprises 13 items rated on a 5-point Likert scale (0=“not at all true*”* to 4=“very true*”*). A total score is obtained by summing the scores for all items. Higher scores indicate higher levels of catastrophic thinking.

#### Pain Intensity

Patients provide their pain rating when they complete their daily diary (Textbox 1) at the same scheduled time using a standard 11-point visual analog scale (0=“no pain” to 10=“most pain possible”) [60]. Average pain intensity ratings are calculated for 7 days before the first treatment session (baseline average pain) and 7 days before the discharge assessment (discharge average pain).

**Daily diary items.**

**Engagement/fun**

- I enjoyed my PT exercises
- How much *fun* did you have during your PT exercises?

**Self-efficacy/confidence**

- While doing your PT exercises, how *strong* did your body feel?
- While doing your PT exercises, how *easy and free* did your movement *feel*?
- While doing your PT exercises, how *worried* were you about *damaging* your body?
- *How confident* did you feel about *playing and doing physical things* after your PT?

**Immersion/distraction**

- I forgot everything around me during my PT exercises.
- How much *time* did you spend thinking about your pain during your PT exercises?

**Lower Extremity Functional Scale/Upper Extremity Functional Index**

- Today, because of my pain, I have ________ difficulty doing my usual work, chores, or school activities.
- Today, because of my pain, I have ________ difficulty doing my usual hobbies, recreational or sporting activities.
- Today, because of my pain, I have ________ difficulty going up or down 10 stairs (about 1 flight of stairs).
- Today, because of my pain, I have ________ difficulty lifting an object, like a bag of groceries, above my head.

**Pain**

- On a scale of 0 (no pain) to 10 (worst possible pain), tell us *how much pain you are feeling right now*.

**Notable events**

- Please make note of anything exciting or stressful that happened today.

**Sleep**

- What time did you get into bed last night?
- What time did you get out of bed this morning?
- How well did you sleep last night?

**PT**

- Did you have a physical therapy appointment today?
- How much time did you spend on your home exercise program today?
- Did you use VR today?

#### Self-efficacy

The Pain Self-Efficacy Scale-Children is a 7-item self-report questionnaire that measures patient beliefs about their ability to complete daily activities despite being in pain [61]. The Pain Self-Efficacy Scale-Children is scored on a 5-point Likert scale whereby patients rate their certainty about their ability to complete an activity (1=“very sure” to 5=“very unsure”), with higher scores indicating less self-efficacy in the context of pain.

#### Patients’ Global Impression of Change

The self-report measure Patients’ Global Impression of Change reflects a patient’s belief about the efficacy of treatment [62]. The Patients’ Global Impression of Change is a 7-point scale depicting a patient’s rating of overall improvement. Patients rate their change as “very much improved,” “much improved,” “minimally improved,” “no change,” “minimally worse,” “much worse,” or “very much worse.”

#### VR Immersion

The child presence measure assesses the patients’ perceived involvement or immersion, realism, and transportation while using the VR headset [63,64]. The measure comprises 12 items rated on a 3-point Likert scale (0=“no” to 2=“a lot*”*). A total score is obtained by summing the scores for all items. Higher scores indicate higher levels of immersion and engagement.

### SCED Outcomes

The participant daily diary consists of 20 items assessing engagement or fun, self-efficacy, immersion or distraction, function, pain, notable events, and sleep (Textbox 1). Daily diaries are collected via LifeData, an app that collects in-the-moment data from study participants by delivering push notifications to participants’ smartphones [42].

### Exploratory Outcomes

#### Modified Borg Dyspnea Scale

The Modified Borg Dyspnea Scale is completed by the patient after each appointment [65]. The Modified Borg Dyspnea Scale measures patient rate of perceived exertion to monitor and guide exercise intensity. The scale item states the following—“How much difficulty is your breathing causing you right now?”—and was slightly modified given the deliverance to state “How much difficulty did the patient’s breathing cause them during today’s therapy session?” Responses are provided on a 12-point scale (0=“nothing at all,” 0.5=“very, very slight (just noticeable),” 5=“severe,” and 10=“maximal”).

#### Physical Assessment

**Figure 3:**
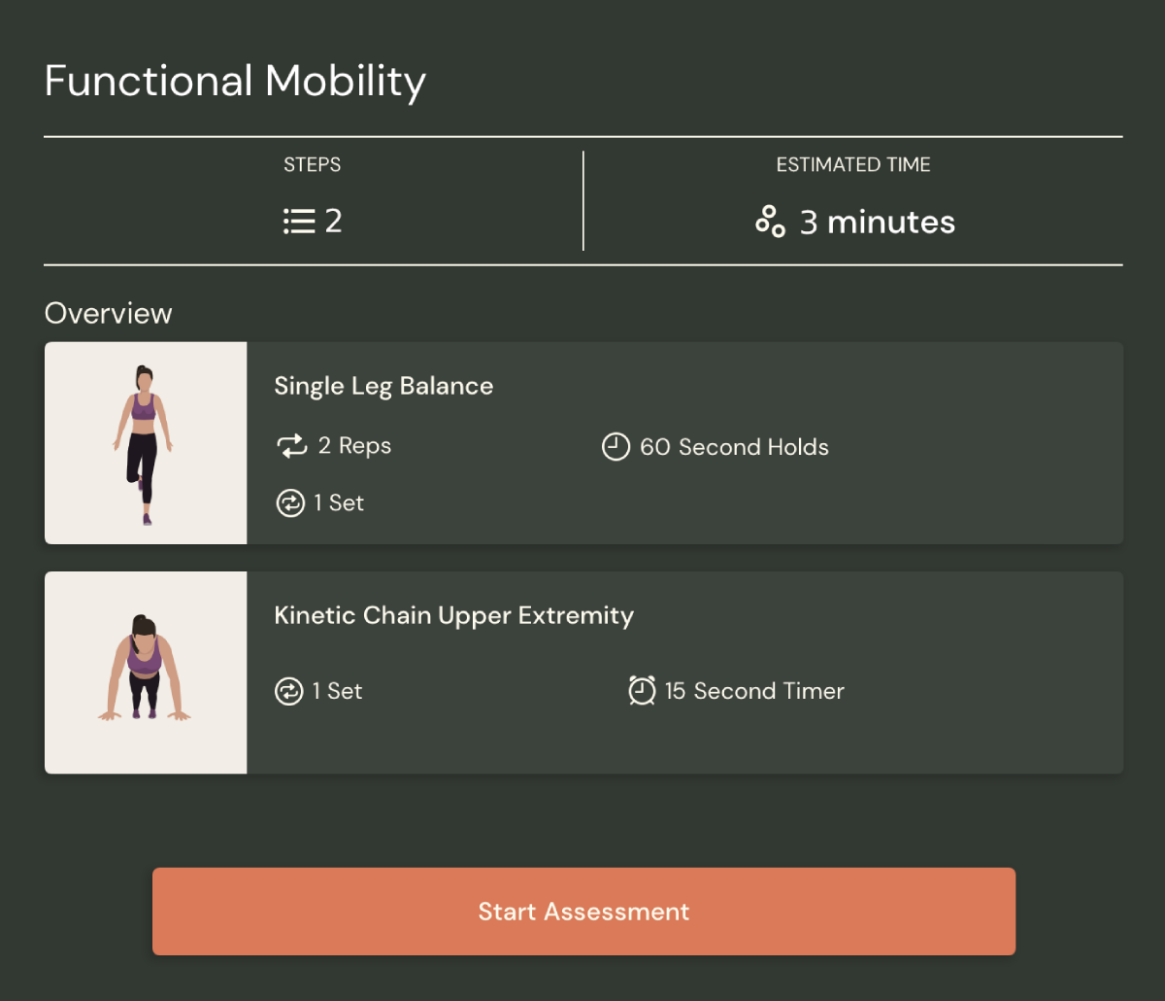
ViFive (ViFive, Inc) app screen showing 2 assessments delivered and captured by ViFive.

To assess changes in physical ability and function, the 6-minute Walk Test [66], Single Leg Balance Test [67], and Closed Kinetic Chain Upper Extremity Stability Test [68] are performed at baseline and discharge. For the 6-minute Walk Test, patients walk back and forth in a straight line for 6 minutes, and the total distance covered is calculated. The Single Leg Balance Test consists of balancing on one leg at a time, and participants’ scores reflect the amount of time they are able to balance within a 60-second time frame. The Closed Kinetic Chain Upper Extremity Stability Test assesses participants’ upper body and core strength by asking them to start on their hands and knees or in a plank position and, while remaining in good form, continually tap the opposite hand that remains planted on the ground. The total score is the number of hand touches completed within the allotted time of 15 seconds. The Walk Test is recorded by a physiotherapist and research assistant. For the remaining 2 exercises, ViFive is used, a motion capture app that reads the patient’s body and allows for automatized counting and timing (Figure 3). The ViFive technology can track additional informatics such as range of motion, balance, flexibility, and endurance metrics as well as real-time pose correction to the user. For example, ViFive captures the patient’s body position during their Closed Kinetic Chain Upper Extremity Stability Test. Changes in performance from baseline to discharge across all 3 tests are assessed.

#### Physical Activity

To examine daily physical activity, the participants wear an ActiGraph watch for the duration of their study participation (baseline phase and across treatment). The ActiLife software (ActiGraph, LLC) will be used to extract data and calculate the mean and peak daily activity. Physical activity (mean and peak) is modeled at 2 time points, and the rate of change in physical activity from baseline to discharge will also be examined.

#### Health Cost Diary

Diaries on health care service use; personal costs; and support provided by family, friends, and professional carers are completed by parents once at baseline, on a weekly basis from pretreatment baseline to end of treatment, and once at the 3-month follow-up. Parents report on youths’ health care service use—general and specialist medical practitioners, physiotherapists, alternative health care practitioners, medications, hospital admissions, and out-of-pocket costs—and other impacts on youth and parental activity—athletic extracurricular activities and parental days off work and sick leave.

### Covariates

#### Medical History

Variables related to chronic pain, including pain onset, duration, and intensity of pain symptoms as well as course and medications, are collected.

#### Demographics

Demographic variables—namely, age, gender, sex, school grade, and ethnicity—are assessed via adolescent and parent reports at baseline.

### Data Analysis

The study biostatisticians conduct all analyses. Covariates (age, pain variables, gender, and diagnosis) are examined for the primary, secondary, additional, and exploratory outcomes.

#### Primary, Secondary, and Additional Outcomes

Linear mixed effects models will be used to compare physiorehabilitation with VR with standard PT across all non-SCED outcomes. We will model our outcomes at 3 time points using a mixed effects linear model with fixed effects for treatment assignment, period, interaction between treatment and period, and baseline covariates, and a random effect for individual. The random effect will allow us to account for the correlation in the outcome within an individual over time.

#### Exploratory Outcomes

To examine biomechanical data, physical assessment metrics will be extracted. We will model physical assessment metrics of walk distance, single leg balance duration, and hand taps using mixed 2 (time)×2 (group) ANOVAs. If physical assessment metrics differ by pain site (upper, trunk, lower, or diffuse), this will be included as a covariate. To examine actigraphy data, the ActiLife software will be used to extract data and calculate the mean and peak daily activity. Published data reduction methods will be used [69]. We will model physical activity (mean and peak) at 2 time points using mixed 2 (time)×2 (group) ANOVAs. The rate of change in physical activity from baseline to discharge will also be examined using the randomization tests used for SCED outcomes described in the following sections. To examine health cost diary data, we will model health care cost variables using *t* tests and linear and mixed regression models.

#### Feasibility Outcomes

Mean satisfaction and acceptability scores will be examined for both patients and clinicians. To assess patient engagement, the mean adolescent daily diary completion, percentage of patient dropouts before treatment completion, and percentage of sessions with benchmark VR met will also be examined. To assess patient and clinician feedback regarding the feasibility and acceptability of the VR treatment, thematic analysis will be conducted of semistructured interviews to identify barriers, catalysts, and perceptions of integrating VR into clinical care. To evaluate engagement in the VR treatment, the mean clinician ratings on the Pittsburgh Rehabilitation Participation Scale will be examined. Acceptability of the VR treatment will be assessed using patient-reported treatment expectancy mean scores. Finally, adherence to the suggested benchmark VR engagement will be assessed by examining the mean time engaged in VR across participations (standard PT and physiorehabilitation with VR) as well as across VR game type (eg, Fruity Feet vs Vacation Simulator).

#### SCED Analyses

The data obtained from the randomized SCED used in this study have a hierarchical 2-level structure with observations (level 1) nested within patients (level 2). This nested structure induces dependency within the data—observations vary not only because of random sampling within a patient but also between different patients. For data analysis, we will use a *hierarchical linear model*, allowing us to combine all patients’ data into a single multilevel model while also considering both the within- and between-patient dependencies. The within- and between-patient variability, as well as the overall effects of the treatment across patients, will be modeled. For conducting the multilevel analysis and obtaining inference results in R (R Foundation for Statistical Computing), MultiSCED will be used [70]. These daily individual data also allow for the use of randomization tests to assess the difference in daily diaries between baseline and discharge and between baseline and 3-month follow-up.

### Sample Size and Power Analysis

The largest feasible sample size will be recruited to obtain as precise estimates as possible of improvement in adolescent function while also ensuring adequate power for the treatment difference in improvement in our primary outcome, physical function. In our primary power calculation, we assumed that we would observe a (medium) effect size of 0.70 for the effect of treatment on outcome (LEFS or UEFI), which corresponds to an absolute difference between physiorehabilitation with VR and standard PT at discharge of 11.9 points assuming an SD of 17 from the validation cohort. This difference of 11.9 equates to 1.32 times the minimal clinically important difference (9). Under this scenario, and accounting for a 20% attrition rate based on previous experience, we will have a power of 80% with 68 participants (34 in each arm) at follow-up. Under more conservative assumptions, we will have a power of 80% with 40 participants (20 in each arm) at follow-up to detect an effect size of ≥0.90. For our secondary outcome, pain-related fear (FOPQ-SF), with 20 participants in each arm, we would have 80% power to detect a minimal clinically important difference between groups (8.6). A recent interoceptive exposure treatment for youth with chronic pain showed improvement in pain-related fear, with a medium effect (Cohen *d*=0.73), suggesting a sample size of 31 per group to achieve 80% power, suitably within the range of these estimates.

### Monitoring

The study is monitored by Navitas Clinical Research for the executive secretary of the National Institute of Arthritis and Musculoskeletal and Skin Diseases. A safety monitoring committee of 3 experts, approved by the National Institute of Arthritis and Musculoskeletal and Skin Diseases via Navitas Clinical Research, meets quarterly to review overall participant enrollment status, accrual, adherence, protocol deviations, and adverse events.

## Results

The physiorehabilitation with VR RCT was prospectively registered at ClinicalTrials.gov (NCT04636177). Analysis of results from the main clinical trial will begin as recruitment progresses, and results are expected in early 2024.

## Discussion

### Overview

Improving treatment outcomes for adolescents with chronic pain requires engagement in gold-standard treatment and notably progressive physical activity as guided by a physiotherapist. Given existing barriers to engaging in this treatment, innovative and engaging technologies may offer an important option for improving engagement and reducing fear and avoidance of pain, thus allowing for the optimal benefit of PT support. A critical element of engagement in PT is the HEP, which requires patients and families to be diligent in completing daily stretching as well as strength and endurance training, all of which can be difficult and uncomfortable and even more so in the context of chronic pain. Improving engagement in PT as well as adherence to at-home exercise programs are important opportunities for potentially accelerating improvements in PT.

Although we know that the use of VR equipment can be helpful in several contexts, we still do not know if it can facilitate improved functioning and reduced pain-related fear and avoidance in the context of PT in a pediatric pain population. These results will add to this growing body of literature by providing a rigorous assessment of the feasibility of physiorehabilitation with VR in outpatient PT for MSK pain and, thus, support or refute the feasibility of disseminating physiorehabilitation with VR for large-scale implementation.

The findings of this study will also illuminate the feasibility of integrating VR technology into current clinical practice across diverse clinical settings, from private to academic medical PT. Engagement and feasibility outcomes will support the understanding of the feasibility of implementing VR within the PT session as well as how VR can augment HEPs for adolescents. Qualitative interview results will further the existing literature [37] in identifying barriers and catalysts to initiating implementation of VR in practice from the perspective of clinicians responsible for intervention implementation.

The addition of the SCED will further highlight the potential of VR to operate as a tailored treatment intervention through the identification of individual experiences and outcomes associated with the use of VR in physiotherapy. Through analysis of individual daily diary data, these findings will support a greater understanding of what elements of the intervention are most impactful and how that may differ across individuals engaged in the study as well as when inclusion of the VR intervention may be most helpful during PT for MSK pain. Together, the results of this RCT, including the SCED and feasibility elements, may support a large hybrid effectiveness-dissemination RCT serving as the basis for potential large-scale implementation of physiorehabilitation with VR and ultimately expand effective, tailored treatment options for adolescents struggling with persistent MSK pain and related fear and disability.

### Study Strengths

This study has several strengths. This is the first pragmatic trial to implement VR in busy and diverse clinical settings, including academic medicine at a major children’s hospital as well as private PT clinics. This offers important information regarding the feasibility of VR in distinct real-world care settings. The embedded single-case design within the RCT study is also a strength as it allows for the evaluation of individual treatment responses (responder or nonresponder) within a small cohort of individuals.

### Study Limitations

With regard to limitations, an emphasis on integration into the flow of clinical care may result in less control in the implementation of the intervention and, thus, create variability in the VR dose. Importantly, we have metrics to assess the degree of use for each participant so that this factor can be adequately accounted for in the analysis. This study is also being implemented within primarily private clinical settings and, thus, generalizability to other settings is limited. Future research should examine the utility of VR in physical therapy settings in more diverse clinical contexts and geographical locations and in populations of diverse patients. Finally, this study relies on the researchers’ ability to engage and support implementation in the care setting, and ongoing evaluation of integration is warranted following the end of the trial as clinics may require the support of the research team to maintain engagement.

## Abbreviations

FDI: Functional Disability Inventory
FOPQ-SF: Fear of Pain Questionnaire-Short Form
HEP: home exercise program
IRB: Institutional Review Board
LEFS: Lower Extremity Functional Scale
MSK: musculoskeletal
PHODA-Youth: Photographs of Daily Activities for Youth
PROMIS: Patient-Reported Outcomes Measurement Information System
PT: physiotherapy
RCT: randomized controlled trial
SCED: single-case experimental design
TEC-C: child Treatment Expectancy and Credibility
TSK-17: 17-item Tampa Scale for Kinesiophobia
UEFI: Upper Extremity Functional Index
VR: virtual reality

## Data Availability

The information and mentioned materials are available from the corresponding author upon reasonable request. Once data are collected and analyzed, they will be posted on ClinicalTrials.gov and will be available from the corresponding author upon reasonable request.
